# Genetic studies of various *Prosopis* species (Leguminosae, Section *Algarobia*) co‐occurring in oases of the Atacama Desert (northern Chile)

**DOI:** 10.1002/ece3.7212

**Published:** 2021-02-10

**Authors:** Cecilia Bessega, Carolina Pometti, Reneé Fortunato, Francisca Greene, Calogero M. Santoro, Virginia McRostie

**Affiliations:** ^1^ Departamento Ecología, Genética y Evolución (EGE), Genética de Especies Leñosas (GEEL) Facultad de Ciencias Exactas y Naturales Universidad de Buenos Aires Buenos Aires Argentina; ^2^ Instituto de Ecología, Genética y Evolución (IEGEBA) CONICET‐Universidad de Buenos Aires Buenos Aires Argentina; ^3^ Instituto de Recursos Biológicos CIRN INTA CONICET Buenos Aires Argentina; ^4^ ESIIyCA Universidad de Morón Morón Argentina; ^5^ San Pedro De Atacama Chile; ^6^ Instituto de Alta Investigación Universidad de Tarapacá Arica Chile; ^7^ Facultad de Ciencias Sociales Escuela de Antropología Pontificia Universidad Católica de Chile Santiago Chile; ^8^ Centro del Desierto de Atacama UC Santiago Chile

**Keywords:** *Algarrobo*, *Chilenses*, genetic diversity, microsatellites, sociocultural factors, structure

## Abstract

In the Atacama Desert from northern Chile (19–24°S), *Prosopis* (Leguminosae) individuals are restricted to oases that are unevenly distributed and isolated from each other by large stretches of barren landscape constituting an interesting study model as the degree of connectivity between natural populations depends on their dispersal capacity and the barriers imposed by the landscape. Our goal was to assess the genetic diversity and the degree of differentiation among groups of *Prosopis* individuals of different species from Section *Algarobia* and putative hybrids (hereafter populations) co‐occurring in these isolated oases from the Atacama Desert and determine whether genetic patterns are associated with dispersal barriers. Thirteen populations were sampled from oases located on three hydrographic basins (Pampa del Tamarugal, Rio Loa, and Salar de Atacama; northern, central, and southern basins, respectively). Individuals genotyped by eight SSRs show high levels of genetic diversity (*H*_O_ = 0.61, *A*_r_ = 3.5) and low but significant genetic differentiation among populations (*F*_ST_ = 0.128, *F*_ST‐ENA_ = 0.129, *D*_JOST_ = 0.238). The AMOVA indicates that most of the variation occurs within individuals (79%) and from the variance among individuals (21%); almost, the same variation can be found between basins and between populations within basins. Differentiation and structure results were not associated with the basins, retrieving up to four genetic clusters and certain admixture in the central populations. Pairwise differentiation comparisons among populations showed inconsistencies considering their distribution throughout the basins. Genetic and geographic distances were significantly correlated at global and within the basins considered (*p* < .02), but low correlation indices were obtained (*r* < .37). These results are discussed in relation to the fragmented landscape, considering both natural and non‐natural (humans) dispersal agents that may be moving *Prosopis* in the Atacama Desert.

## Graphical abstract

We assess the genetic diversity and the degree of differentiation among groups of *Prosopis* individuals of different species from Section *Algarobia* and putative hybrids co‐occurring in isolated oases from the Atacama Desert (19–24°S) in Chile. Genetic results are discussed in relation to the fragmented landscape, considering both natural and non‐natural (humans) dispersal agents that may be moving *Prosopis*in the desert.

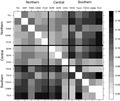

## 1 INTRODUCTION

The Atacama Desert in northern Chile extends for more than 1,000 km along the western coast of South America and is one of the most arid deserts on Earth. The extreme aridity is primarily caused by the cold water of the Humboldt Current, running parallel to the Chilean and southern Peruvian coasts and preventing precipitation in the coastal areas. Moreover, aridity is intensified by the Andes cord that produces a rain‐shadow effect, blocking moisture from the Amazon basin (Houston & Hartley, 2003). However, during summer some of this moisture passes over the Andes, intensifying discharges of water flows through deeply incised gorges and shallow riparian and underground water systems (Jayne et al., 2016; Vuille et al., 2012).

In the Atacama Desert, within 19–24°S, three hydrological basins can be recognized: Pampa del Tamarugal, Loa River, and Salar de Atacama (Mortimer, 1980; Nester et al., 2007). In Pampa del Tamarugal or northern basin, the vegetation corresponds to the “Desierto del Tamarugal” (Gajardo, 1994) and is distributed in areas where the water table is relatively shallow or receives riparian flows originated in the Andes by summer precipitations (Barros, 2010). This basin has evidence of several human settlements since the Late Pleistocene, and it is connected by a small ravine with the lower course of the Loa River, nearby its mouth in the Pacific Coast (Nester et al., 2007; Pfeiffer et al., 2018). The Loa River or the central basin is the only exoreic stream traversing the desert from the Andes to the Pacific Ocean. It is placed between the Pampa del Tamarugal to the north and the Salar de Atacama to the southeast. This basin was used as a natural and cultural corridor since pre‐Columbian times (Marquet et al., 1998; Núñez, 1971), passing beyond different vegetation belts, although riparian plants predominate along the watercourse (Gajardo, 1994; Villagrán & Castro, 2004). Finally, the Salar de Atacama is an endorheic basin within the Prepuna foothills (Arriagada et al., 2006; Marazuela et al., 2019). Different quebradas flow into this system giving rise to several oases with riparian vegetation (Gajardo, 1994; Villagrán & Castro, 2004). This area was also key in the history of pre‐European societies (Núñez & Santoro, 1988).

The genus *Prosopis* (Leguminosae) includes 44 species distributed in Southwest Asia, Africa, and predominantly America (Burkart, 1976). *Prosopis* species are important plants in arid and semiarid regions, and many species from one particular Section*, Algarobia (algarrobo)*, are considered multipurpose trees. From an economic perspective, humans use it as food as the raw pods can be eaten directly or processed within a variety of culinary preparations (Capparelli, 2007). Their pods and leaves provide forage for animals, and their flowers are melliferous. The wood is useful for diverse manufactures, having higher caloric value than other sympatric species (Burkart, 1976; Cony, 1996; Roig, 1993; Villagrán & Castro, 2004). From an ecological view, they can grow on sandy soils and contribute to stabilize dunes, combat desertification, and reforest degraded areas (Bessega et al., 2019; Burkart, 1976; Cony, 1996; Roig, 1993).

**FIGURE 1:**
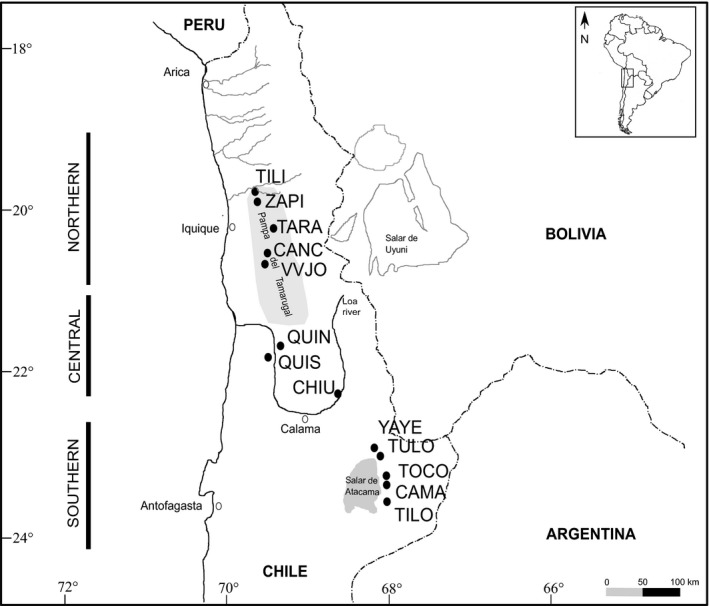
Ecosystems and areas of sampling sites across the Atacama Desert (Chile). TILI: Tiliviche, ZAPI: Zapiga, TARA: Tarapacá, CANC: Canchones, VVJO: Valle Viejo, QUIN: Quillagua Norte, QUIS: Quillagua Sur, CHIU: Chiu‐Chiu, YAYE: Yaye, TULO: Tulor, TOCO: Toconao, CAMA: Camar, TILO: Tilomonte

In Chile, according to Burkart (1976) six species from Section *Strombocarpa* and *Algarobia* can be found (*P. strombulifera*, *P. burkartii*, *P. tamarugo*, *P. chilensis*, *P. flexuosa*, and *P. alba*), but Muñoz (1981) also cited *P. nigra* for the north of Chile. While the Pampa del Tamarugal forest is dominated by three species from Section *Strombocarpa* (*P. burkartii*, *P. strombulifera*, and the endemic *P. tamarugo*), individuals belonging to different species from Section *Algarobia* (*P. alba*, *P. flexuosa*, and *P. chilensis*) can be found up to 3,000 masl and are distributed from the Pampa del Tamarugal to the Salar de Atacama basin (Figure 1; Barros, 2010; Carevic et al., 2012). Natural *algarrobo* populations are restricted to oases unevenly distributed and isolated from each other by large stretches of barren landscape. This area is categorized as an absolute desert since plant life is practically absent in much of its extension (Gajardo, 1994; Gayo, Latorre, Jordan, et al., 2012; Gayo et al., 2012; Latorre et al., 2002, 2005).

The effects produced by landscape isolation may be comparable with those occurring on forest populations due to urbanization, overexploitation, and land conversion into crop plantations, in terms of habitat fragmentation, mating system changes, and gene flow restriction (Cascante et al., 2002; Jump & Penuelas, 2006). When populations become genetically isolated, they are at risk of losing the genetic diversity that is critical to their long‐term survival (Sork & Smouse, 2006). As a consequence of the isolation, an immediate loss of alleles due to the reduction in the population is expected, with the consequence of inbreeding, population divergence increase, and genetic diversity reduction within population patches (Lowe et al., 2005). However, the longevity of the trees and effective seed and pollen dispersal (when possible) can enhance their resistance to the negative effect of the forest fragmentation (Jump & Penuelas, 2006).

In trees, gene flow is mediated by mobile structures with reproductive function as seeds, pollen, and vegetative propagules, while the adult stage is sessile. Limitation in the dispersal distance of seeds and/or pollen over time will produce a particular genetic structure consisting of decreasing relatedness of individuals with increasing geographical distance (Loiselle et al., 1995). The natural *Prosopis* populations in Atacama Desert constitute an interesting study model as the degree of connectivity between populations depends on their dispersal capacity and the barriers imposed by the landscape. *Prosopis* populations are expected to be structured because pollen and seed dispersals are limited (Bessega, Ferreyra, Julio, et al., 2000; Bessega et al., 2011, 2017). The endozoic seed dispersal determines that seeds from the same mother plant are eaten by small‐ and medium‐sized herbivores (Reynolds, 1954) and transported away jointly. They are then deposited in dung, and full‐ or half‐sib seeds tend to germinate together in a narrow area. Besides, the pollination is entomophilous (Genisse et al., 1990) favoring crosses among near neighbor plants.

The main objective of this study was to assess the genetic diversity and the degree of differentiation among groups of *Prosopis* individuals of different species from Section *Algarobia* and putative hybrids (hereafter populations) co‐occurring in the isolated oases from the Atacama Desert and determine whether genetic patterns are related to dispersal barriers. For this, we characterized by microsatellites the variability, genetic differentiation, and genetic structure among 13 populations considering three distant basins. Through these analyses, we addressed the following questions: (a) Are there differences in the genetic diversity parameters among populations? (b) How is the genetic variation distributed considering the different hierarchical levels (basins, populations and individuals)? (c) What is the level of differentiation and gene flow among populations? (d) Is there a genetic pattern compatible with isolation by distance (IBD)? (e) Is there genetic structure evidence? As the Atacama Desert provides habitat fragmentation that could affect the natural gene flow, given by the large stretches of hyperarid landscape, we discuss the genetic results considering the dispersal barriers that separate the populations.

Given that *Prosopis* populations are restricted to isolated oases, the current hypothesis is that the gene flow restrictions among different populations would determine significant genetic differentiation among populations. Additionally, the dispersal limitation described for *Prosopis* species (Bessega, Ferreyra, Julio, et al., 2000; Bessega et al., 2011, 2017) and the barriers imposed by the landscape should be reflected in population genetic structure.

## 2 MATERIALS AND METHODS

### 2.1 Study sites and sampling

The sampled range in our study extends from 19 to 24°S (Figure 1), within the core of the Atacama Desert and encompasses three hydrological systems: (a) the northern basin or inland basin of Pampa del Tamarugal (19.5–20.76°S), close to the Pacific coast; (b) the central basin or the Loa River, Calama area (21.6–22.3°S); and (c) the southern basin or Salar de Atacama (22.9–23.8°S), at the slope of the Andes and far away from the Pacific Coast. Within the Pampa del Tamarugal basin, there are 325 km^2^ of *P*. *alba* populations planted by the Chilean State during the middle of the 20th century that we did not sample as natural populations were our study goal (Barros, 2010).

We sampled thirteen natural populations of *Prosopis* species from Section *Algarobia* (Table 1; Figure 1). We collected fresh young leaves and pods for species identification. Each sampled tree was deposited at the INTA, Hurlingham BAB herbarium, Buenos Aires, Argentina. Species identifications were done considering leaves, spines, fruits, and tree form following Burkart (1976), Burkart and Simpson (1977), and Palacios and Brizuela (2005) proposals. For each sample, we had considered habit form, leaf division, leaflets (length, width, distance between leaflets), spines (types and length), and fruits (shape, length, and epicarp color). Likewise, the identification was corroborated with the type specimen of the taxa available at https://plants.jstor.org/. As the identification of hybrids was based on morphological traits, not confirmed by molecular data, the term “putative hybrid” is used throughout the manuscript. Following Vilardi et al. (1988) method, which recommends sampling trees separated more than 30–50 m from each other to avoid collecting genetically related material, we did not gather all the trees present in each oasis, but the maximum possible. Six to seventeen adult trees (diameter at breast height [DBH] > 80 cm) were sampled in each location because populations were small, giving a total of 126 adult individuals. Geographical coordinates were recorded for each sampled tree using GPS Garmin Etrex 20, datum WGS84.

**TABLE 1:** Locality, acronyms, geographic coordinates (latitude, longitude, altitude), number of samples (N), basin, taxonomic classification of the sampled material, and herbarium voucher numbers (Voucher No) of the thirteen *Prosopis* populations (Pop) sampled in the Atacama Desert (Chile)

Pop	Locality	Acronyms	Latitude (degrees)	Longitude (degrees)	Altitude (masl)	*N*	Basin	Sampled material (*n*)	Voucher no
1	Tiliviche	TILI	−19.55308	−69.95375	1,011	14	N	*P. a* (7), *P. fle* (7)	13409–13421
2	Zapiga	ZAPI	−19.63436	−69.95222	1,112	17	N	*P. a* (2), *P. chi* (1), *P. fle* (10), *P. nig* (3), *P. a* x *P. fle* (1)	13223–13239
3	Tarapacá	TARA	−19.90145	−69.48397	1,465	8	N	*P. chi* (5), *P. fle* (3)	13246/13249, 13253, 13255/13260, 13262, 13263–13268–13272, 13275/13380
4	Canchones	CANC	−20.43496	−69.53340	980	11	N	*P. a* (5), *P. chi* (6)	13260, 13262–13263, 13271–13272, 13275–13280
5	Victoria Viejo	VVJO	−20.76900	−69.56855	962	6	N	*P. a* (3), *P. chi* (3)	13288–13302
6	Quillagua Norte	QUIN	−21.61749	−69.55209	776	6	C	*P. fle* (1), *P. chi* (2), *P. nig* (2) *P. chi* x *P. fle* (1)	13304–13308
7	Quillagua Sur	QUIS	−21.65166	−69.53409	804	6	C	*P. fle* (5), *P. fle* x *P. chi* (1)	13313–13318
8	Chiu‐Chiu	CHIU	−22.31751	−68.65020	2,545	10	C	*P. a* (4), *P. chi* (6)	13325, 13328, 13331/32, 13335, 1338–13342
9	Yaye	YAYE	−22.91853	−68.20203	2,415	8	S	*P. a* (4), *P. fle* (3), *P. a* x *P. chi* (1)	13347/13351, 1353, 1356, 13359/60
10	Tulor	TULO	−22.96842	−68.21942	2,394	6	S	*P. fle* (5), *P. fle* x *P. chi* (1)	13633–13638
11	Toconao	TOCO	−23.18508	−68.00165	2,511	8	S	*P. a* (6), *P. chi* (2)	13405, 13607, 13618–13623
12	Camar	CAMA	−23.40098	−67.95119	2,726	10	S	*P. chi* (4), *P. fle* (5), *P. chi* x *P. fle* (1)	13393–12402
13	Tilomonte	TILO	−23.78556	−68.10135	2,377	16	S	*P. a* (1), *P. chi* (8), *P. fle* (6), *P. chi* x *P. fle* (1)	13367–13382

Abbreviations: N, northern; C, central; S, southern; masl, meters above sea level; *n*, sample size; *P. a*, *P. alba*; *P. fle*, *P. flexuosa*; *P. chi*, *P. chilensis*; *P. nig*, *P. nigra*.

### 2.2 Microsatellite analysis

Total DNA was isolated from leaf material using DNA easy Plant Mini kit (Qiagen Inc.). We used a total of eight microsatellites; four developed by Mottura et al. (2005) for *P. chilensis* and *P. flexuosa*: Mo08, Mo09, Mo05, and Mo13, and four developed by Bessega et al. (2013) for *P. alba* and *P. chilensis*: GL9, GL12, GL8, and GL21. The SSRs were amplified using forward primers labeled with a fluorescent dye (6‐FAM and HEX, Invitrogen). The PCR amplifications were carried out in a 50 µl reaction volume containing 10–30 ng DNA, 0.6 µM each primer, 0.2 mM dNTPs, 0.3 U Taq DNA polymerase (Invitrogen), and 1.5 mM MgCl_2_. A T100 Thermal Cycler (Life Science Research, Bio‐Rad) was used for amplifications with a cycling profile of initial denaturation at 94°C for 5 min followed by 35 cycles at 94° for 45 s denaturation, primer‐specific annealing temperature (56°–59°) for 45 s and at 72°C for 45 s extension, and a final extension step at 72° for 10 min. PCR products were electrophoresed using Macrogen service (www.dna.macrogen.com) and sized using GENEMARKER ver 1.91 (SoftGenetics, 2019).

### 2.3 Data analysis

#### 2.3.1 Genetic diversity and inbreeding coefficient *F*_IS_

Linkage disequilibrium was tested by the index of association (*I*_A_) and a slightly modified statistic which is independent of the number of *loci* (*r*_d_) (Agapow & Burt, 2001). *I*_A_ and *r*_d_ coefficients, with 9,999 randomizations, were estimated with the package *poppr* (Kamvar et al., 2014, 2015) of R program ver. 4.0.1 (R Development Core Team, 2020).

Genetic diversity was quantified in each population through the mean number of observed alleles per locus (*A*), mean percentage of total alleles observed per locus per population (%TA), allelic richness (*A*_r_), and observed heterozygosity (*H*_O_) and expected heterozygosity (*H*_E_) under the Hardy–Weinberg equilibrium. Genetic variability estimate mean was compared among populations by the nonparametric ANOVA test (Kruskal–Wallis test). Homozygote excess/deficiency was quantified by the fixation index (*F*_IS_) which was considered significant when the zero is not contained in its 95% confidence interval (based on 2,000 bootstrap resampling). *A*, %TA, and *H*_O_ were estimated using the package *diveRsity* (Keenan et al., 2013) of R software; *A*_r_ was estimated using ASDE (Szpiech et al., 2008) that allows the correction for samples bias; and *H*_E_ was estimated using SPAGeDi (Hardy & Vekemans, 2002) that performs the Nei (1978) gene correction for small number of individuals.

#### 2.3.2 Genetic differentiation

From the microsatellite's marker data, three differentiation coefficients were estimated. *F*_ST_ (Wright, 1951) was estimated with the package *hierfstat* (Goudet, 2005) of the software R, and its significance was determined by the *G* test (*test.g* function). In order to avoid bias induced by the presence of null alleles, *F*_ST_ excluding null alleles (*F*_ST‐ENA_) was also estimated using the software FreeNA (Chapuis & Estoup, 2007). *D*_JOST_ (Jost, 2008) and its significance were calculated using 1,000 permutations as implemented in GenAlEx 6.5 (Peakall & Smouse, 2012). *F*_ST_, *F*_ST‐ENA_, and *D*_JOST_ were also estimated within each sampled basins of the Atacama Desert (northern, central, and southern) and also as pairwise population genetic differentiation indices. Heat map of *F*_ST_ pairwise comparisons between populations was conducted using the *levelplot* function of the *lattice* package (Sarkar, 2008) of the software R. Gene flow (*Nm*) was calculated assuming drift–migration equilibrium using the formula: *Nm *= (1 − *F*_ST_)/4*F*_ST_ (Slatkin & Barton, 1989).

Genetic differentiation estimate mean within and among basins was compared by the nonparametric ANOVA test (Kruskal–Wallis test), and pairwise comparisons were performed by Wilcoxon's pairwise tests using, respectively, the functions *kruskal.test* and *pairwise.wilcox.test* of R software.

To estimate the distribution of genetic diversity, the analysis of molecular variance (AMOVA) was assessed using *Φ* statistics by means of the package *poppr* of R. The molecular differentiation was partitioned into among the sampled basins, among populations within these basins, and among individuals within population components. The significance of *Φ* statistics was obtained with the function *randtest.amova* of the *ade4* package (Bougeard & Dray, 2018; Dray & Dufour, 2007; Dray et al., 2007) of R software with 2,000 permutations.

The differentiation among populations was also evaluated by discriminant analysis of principal components (DAPC; Jombart et al., 2010), using the function *dapc* of the *adegenet* package (Jombart, 2008; Jombart & Ahmed, 2011) of software R. This analysis was conducted with *prior* information on individual populations.

#### 2.3.3 Genetic structure among populations

A Bayesian model‐based cluster analysis was performed using the program STRUCTURE version 2.3.4 (Pritchard et al., 2009). We explored which value of *K* maximized the likelihood of the data. The burn‐in period and the number of Markov chain Monte Carlo (MCMC) repetitions were 25,000 and 50,000, respectively, and *K* values were averaged across ten iterations. Considering the range of population distribution, both admixture and no‐admixture models were run considering correlated allele frequencies, no sampling locations as prior information and *K* from 2 to 14. The optimum *K* value was determined with STRUCTURE HARVESTER (Earl & vonHoldt, 2012) inputting 10 runs per *K* by comparing the average log‐likelihood of data for each value of *K* (Pritchard et al., 2000) and by the Δ*K* statistic (Evanno et al., 2005), based on the second‐order rate of change in the log‐likelihood of data between successive *K* values.

#### 2.3.4 Correlation between genetic and geographic distance

In order to test isolation by distance (IBD), two pairwise population distance matrices (genetic distances and geographic distances) were constructed and compared through the *Mantel* tests with 9,999 permutations using the software GenAlEx 6.5 (Peakall & Smouse, 2012). A pairwise, individual‐by‐individual (N × N) genetic distance matrix based on *multilocus* genotypes was calculated (GD). For a single‐locus analysis, with *i*‐th, *j*‐th, *k*‐th, and *i*‐th different alleles, a set of squared distances are defined as d2 (*ii*, *ii*) = 0, d2 (*ij*, *ij*) = 0, d2 (*ii*, *ij*) = 1, d2 (*ij*, *ik*) = 1, d2 (*ij*, *kl*) = 2, d2 (*ii*, *jk*) = 3, and d2 (*ii*, *jj*) = 4 (Peakall et al., 1995; Smouse & Peakall, 1999). Geographic distances were calculated from the coordinates of sampled localities.

## 3 RESULTS

### 3.1 Genetic diversity and inbreeding coefficient *F*_IS_

Individuals sampled were determined as *P. alba*, *P. chilensis*, *P. flexuosa*, and *P. nigra* and just one putative hybrid was identified in ZAPI, QUIN, QUIS, YAYE, TULO, CAMA, and TILO (Table 1).

The analysis of linkage disequilibrium in the whole sample yielded significant results (*p* = .001), which are attributable to four populations (TILI, TARA, CANC, and VVJO). In the other nine populations, the association index was nonsignificant (Table 2); however, after applying Bonferroni's correction for multiple tests, linkage disequilibrium was significant in only one population suggesting that the eight *loci* markers were genetically independent.

**TABLE 2:** Linkage disequilibrium evaluation by index of association *I*_A_ and *r*_d_ in the thirteen oases of *Prosopis* analyzed (Pop)

Pop	*I*_A_	*r*_d_	*P*
TILI	0.361	0.053	.037
ZAPI	0.118	0.017	.178
TARA	0.540	0.078	.015
CANC	0.540	0.078	.013
VVJO	1.515	0.224	.001
QUIN	−0.355	−0.054	.851
QUIS	−0.354	−0.056	.875
CHIU	0.319	0.046	.080
YAYE	−0.157	−0.023	.640
TULO	0.667	0.103	.051
TOCO	−0.603	−0.089	.907
CAMA	0.037	0.055	.404
TILO	−0.177	−0.026	.869
Total	0.281	0.040	.001

Note

*P* = significance of *r*_d_ estimated by 9,999 permutations.

Pop = population acronyms from Table 1.

The eight SSR loci analyzed in the populations were highly variable showing from two to eight alleles per locus. Percentages of total alleles observed per population (%TA), allelic richness (*A*_r_), and heterozygosity (*H*_O_, *H*_E_) were relatively high in all the studied populations (Table 3). Several indices indicated that TULO was the population with the lowest genetic variation and CHIU the most variable. The percentage of total alleles observed per population and allelic richness ranged from 47.8% and 2.7, respectively, in TULO to 64.5% and 4.1, respectively, in CHIU with a mean value of 56.3% for %TA and 3.5 for *A*_r_. The highest value of *H*_E_ was detected in CHIU (*H*_E_ = 0.73) and the lowest in TULO (*H*_E_ = 0.56). However, KW‐ANOVA indicates that the differences among populations are not significant for all the estimates obtained [%TA (*X* ^2^ = 8.43 *p* = .75), *A*_r_ (*X* ^2^ = 17.23 *p* = .14), *H*_O_ (*X* ^2^ = 8.71 *p* = .73), and *H*_E_ (*X* ^2^ = 7.37 *p* = .83)].

**TABLE 3:** Genetic diversity indices in the thirteen *Prosopis* population based on eight SSR loci

	*N*	A (*SD*)	%TA (*SD*)	*A*_r_ (*SD*)	*H*_O_ (*SD*)	*H*_E_ (*SD*)	*F*_IS_	CI_95%_	
TILI	14.000 (0.000)	35.000 (1.598)	55.050 (20.327)	3.838 (1.315)	0.640 (0.224)	0.620 (0.205)	−0.075*	**−0.178**	**−0.003**
ZAPI	17.000 (0.000)	39.000 (1.727)	60.860 (18.195)	4.113 (1.316)	0.540 (0.172)	0.621 (0.163)	0.109*	**0.007**	**0.199**
TARA	8.000 (0.000)	36.000 (1.414)	57.130 (18.561)	3.497 (0.869)	0.580 (0.251)	0.676 (0.120)	0.088	−0.106	0.222
CANC	11.000 (0.000)	39.000 (1.458)	62.350 (20.066)	3.874 (0.988)	0.550 (0.164)	0.656 (0.138)	0.129	−0.049	0.281
VVJO	6.000 (0.000)	31.000 (1.458)	50.070 (22.378)	3.043 (0.772)	0.690 (0.208)	0.686 (0.127)	−0.094	−0.357	0.083
QUIN	6.000 (0.000)	35.000 (1.188)	57.770 (23.025)	3.292 (0.770)	0.750 (0.177)	0.701 (0.154)	−0.168*	**−0.366**	**−0.035**
QUIS	6.000 (0.000)	32.000 (1.309)	52.680 (22.471)	2.999 (0.762)	0.580 (0.235)	0.648 (0.149)	0.018	−0.203	0.176
CHIU	9.880 (0.354)	41.000 (1.642)	64.510 (18.593)	4.128 (1.120)	0.570 (0.209)	0.727 (0.113)	0.177*	**0.006**	**0.304**
YAYE	7.880 (0.354)	32.000 (1.512)	52.050 (22.119)	3.153 (0.884)	0.590 (0.153)	0.624 (0.151)	−0.008	−0.173	0.092
TULO	6.000 (0.000)	29.000 (1.923)	47.780 (26.658)	2.745 (0.975)	0.670 (0.356)	0.563 (0.177)	−0.227*	**−0.539**	**−0.089**
TOCO	8.000 (0.000)	34.000 (1.165)	55.920 (21.537)	3.288 (0.814)	0.620 (0.164)	0.625 (0.167)	−0.067	−0.182	0.016
CAMA	10.000 (0.000)	36.000 (1.852)	57.240 (22.134)	3.537 (1.147)	0.620 (0.243)	0.626 (0.156)	−0.050	−0.213	0.101
TILO	16.000 (0.000)	37.000 (2.134)	58.920 (24.919)	3.716 (1.434)	0.520 (0.286)	0.593 (0.204)	0.040	−0.044	0.114
Mean	**9.674 (6.047)**	**35.077 (3.339)**	**56.333 (4.638)**	**3.479 (0.232)**	**0.609 (0.063)**	**0.643 (0.028)**	**−0.010** (**0.119**)		

Note

*N*, mean number of individuals analyzed per locus; A, observed alleles per locus; %TA, mean percentage of total alleles observed per population; *A*_r_, allelic richness corrected for sample bias (Szpiech et al., 2008); *H*_O_, observed heterozygosity; *H*_E_, expected heterozygosity corrected for sample size (Nei, 1978); *F*_IS_, inbreeding coefficient, *F*_IS_ was considered significant (*) when zero is not included in 95% confidence intervals (CI_95%_). *SD* in parenthesis.

In five populations, *F*_IS_ was significant (according to their CI_95%_; Table 3), and the ratio negative/positive was 2/3, showing no general trend to heterozygote excess or deficiency. Moreover, *F*_IS_ was negative in seven and positive in six populations. This result fits the expectation for *F*_IS_ estimates in populations under the Hardy–Weinberg equilibrium, because when the actual *F*_IS_ equals 0, over‐ and underestimates of *F*_IS_ (due to sampling error) occur with the same frequency.

### 3.2 Genetic differentiation

Analyses based on *F*_ST_, *F*_ST‐ENA_, and *D*_JOST_ indices yielded similar results, indicating that the overall level of genetic differentiation among the thirteen populations was highly significant (*F*_ST_ = 0.128, *p* ≤ .001; *D*_JOST_ = 0.238 *p* ≤ .001; Table 4). Considering all populations, 1.69 migrants per generation were estimated from *F*_ST_ (Table 4). For northern and southern basin populations, the genetic differentiation indices dropped up to near 30% (*F*_ST_ = 0.087 and 0.092, *D*_JOST_ = 0.151 and 0.182, respectively) but remain highly significant. Based on *F*_ST_, the gene flow becomes 2.62 and 2.47 migrants per generation in northern and southern basins, respectively. In the central basin, differentiation was significant according to *F*_ST_ index (*F*_ST_ = 0.035) and gene flow was much higher in comparison with northern and southern basins (*Nm* = 6.95); however, based on *D*_JOST_ the differentiation among populations within this basin was not significant (*D*_JOST_ = 0.066). In reference to the global *F*_ST‐ENA_ estimation (*F*_ST‐ENA_ = 0.129), similar decreases in the genetic differentiation indices were obtained within the three basins (*F*_ST‐ENA_ = 0.088, 0.042, and 0.087 for the northern, central, and southern basins, respectively; Table 4).

**TABLE 4:** Genetic differentiation estimated by *F*_ST_, *F*_ST‐ENA_ *,* and *D*_JOST_ at different geographic levels and gene flow estimation (*Nm*) based on *F*_ST_ index

Level	Populations	*F*_ST_ (*p*)	*Nm*	*F*_ST‐ENA_ (CI_95%_)	*D*_JOST_ (*p*)
Total (13 pops)	TILI, ZAPI, TARA, CANC, VVJO, QUIN, QUIS, CHIU, YAYE, TILO, CAMA, TOCO, TULO	0.128 (≤.001)	1.69	0.129 (0.082–0.191)	0.238 (.001)
Northern (5 pops)	TILI, ZAPI, TARA, CANC, VVJO	0.087 (.001)	2.62	0.088 (0.043–0.156)	0.151 (.001)
Central (3 pops)	QUIN, QUIS, CHIU	0.035 (.020)	6.95	0.042 (0–0.084)	0.066 (.087)
Southern (5 pops)	YAYE, TULO, TOCO, CAMA, TILO	0.092 (.001)	2.47	0.087 (0.059–0.111)	0.182 (.001)

Note

Population abbreviations are as in Table 1, CI_95%_ = 95% confidence intervals.

**FIGURE 2:**
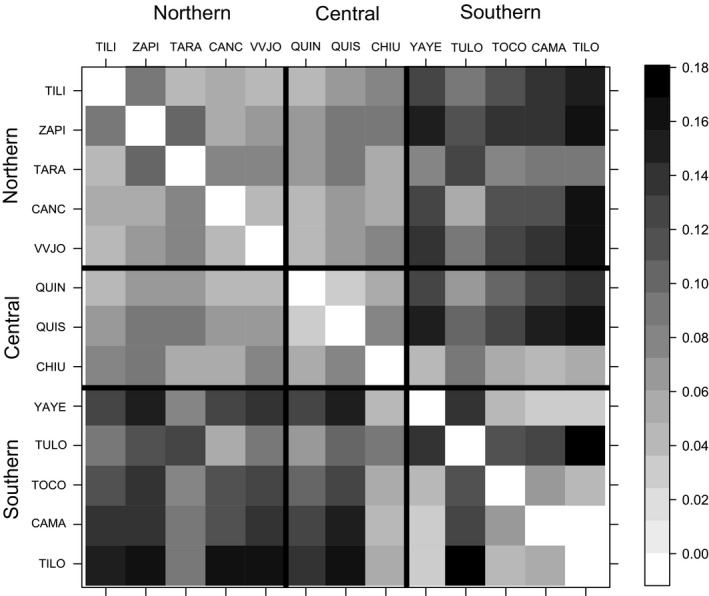
Heat map of *F*_ST_ pairwise comparisons between populations. White cells are low *F*_ST_ values; darker cells are increasingly higher *F*_ST_. TILI: Tiliviche, ZAPI: Zapiga, TARA: Tarapacá, CANC: Canchones, VVJO: Valle Viejo, QUIN: Quillagua Norte, QUIS: Quillagua Sur, CHIU: Chiu‐Chiu, YAYE: Yaye, TULO: Tulor, TOCO: Toconao, CAMA: Camar, TILO: Tilomonte

The genetic differentiation between populations from the same or different geographical basins showed the same tendency by *F*_ST_, *F*_ST‐ENA,_ and *D*_JOST_ (Tables S1‐S3), pairwise genetic differentiation expressed by *F*_ST_ was small to moderate (*F*_ST_ < 0.17), but a trend toward higher differentiation between populations from different basins was observed (Tables S1‐S3; Figure 2). Genetic differentiation between populations within the northern group was low (*F*_ST_ < 0.075) with the exception of ZAPI‐TILI and ZAPI‐TARA comparison that was comparatively higher (*F*_ST_ = 0.096 and 0.108, respectively). In the central group, *F*_ST_ was also very low (*F*_ST_ < 0.078), while the *F*_ST_ between populations of the southern basin was low among almost all the population pairs studied (<0.066). The exception was TULO, which was highly differentiated from all the remaining populations of the southern basin (0.120 > *F*_ST_ < 0.169).

The mean pairwise differentiation estimates among populations from northern, central, and southern basins showed significant variation of *F*_ST_ (*X* ^2^ = 26.59, *p* = 6.83 × 10^−5^), *F*_ST‐ENA_ (*X* ^2^ = 27.81, *p* = 3.97 × 10^−5^), and *D*_JOST_ (*X* ^2^ = 27.40, *p* = 4.75 × 10^−5^). For the three indices, the Wilcoxon pairwise comparisons test was significant among differentiation indices from northern populations with the central and the southern ones (*p* < 5 × 10^−5^ and *p* < .0093, respectively). The *F*_ST_ between populations from northern and central basins were lower in comparison with populations from northern and southern basins. Considering the central and northern basins, the most differentiated population pairs were TARA‐QUIS and ZAPI‐QUIS with *F*_ST_ = 0.093 and 0.092, respectively. The largest *F*_ST_ index obtained from comparing populations from northern and southern basins correspond to the pair ZAPI‐TILO (*F*_ST_ = 0.168).

The AMOVA (Table 5) indicated that the among‐basin differentiation (*Φ*_RT_ = 0.207), the variation among populations within basins (*Φ*_PR_ = 0.068) and among individuals within populations (*Φ*_IP_ = 0.081) were all highly significant (*p* < .0005). The distribution of molecular variance at different hierarchical levels indicated that most of the variation occurs within individuals (79.32%), and consequently, the variance among individuals resulted in 20.68%. Within this level, the between‐basin component (7.40%) was almost equal to the between‐population within‐basin component (7.47%) followed by the between‐individual within population component (5.81%) representing 35.78%, 36.12%, and 28.10%, respectively.

**TABLE 5:** Analysis of molecular variance (AMOVA) based on eight SSR loci considering basins, populations, and individuals

Source of variation	*df*	SS	MS	Est. Var.	%	Φ	*p*
*Between basins*	2	49.43	24.71	0.22	7.40	0.207	.00049
*Between populations within basins*	10	69.59	6.96	0.22	7.47	0.068	.00049
*Between individuals within populations*	113	307.52	2.72	0.17	5.81	0.081	.00049
*Within individuals*	126	299.09	2.37	2.37	79.32	0.074	.00749
Total	251	725.63	2.89	2.99	100		

Note

*df*: degrees of freedom, SS: sum of squares, MS: mean squares, Est Var.: estimated variability, %: proportion of genetic variability, Φ: fixation index, P: significance level.

**FIGURE 3:**
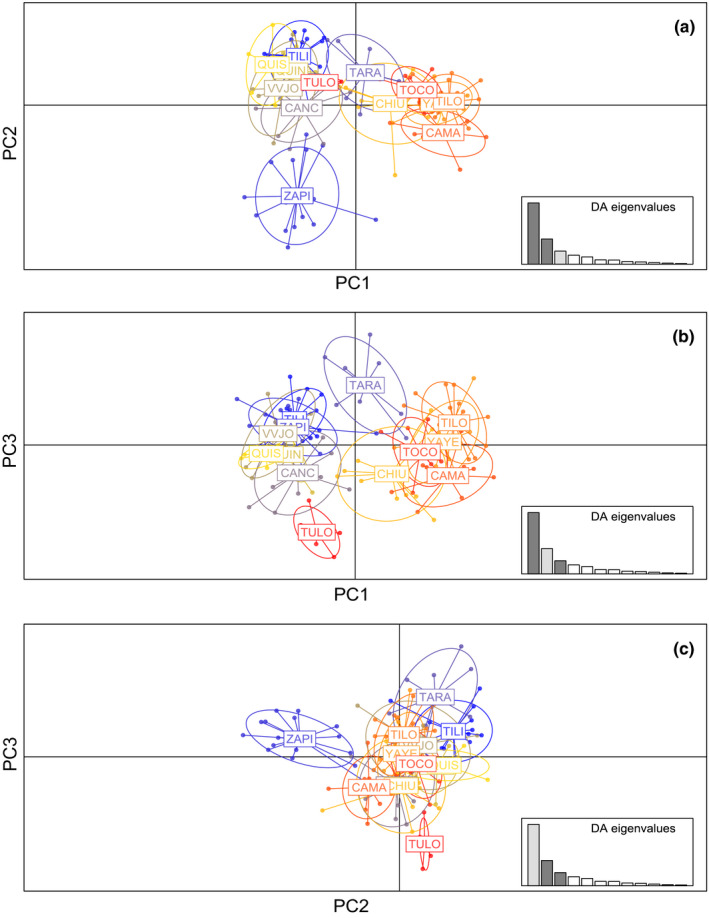
Scatterplot of individuals on the three principal components obtained by discriminant analysis of principal components (*DAPC*). (a) PC1 versus PC2; (b) PC1 versus PC3; and (c) PC2 versus PC3. The graph represents the individuals as dots and the groups as inertia ellipses. TILI: Tiliviche, ZAPI: Zapiga, TARA: Tarapacá, CANC: Canchones, VVJO: Valle Viejo, QUIN: Quillagua Norte, QUIS: Quillagua Sur, CHIU: Chiu‐Chiu, YAYE: Yaye, TULO: Tulor, TOCO: Toconao, CAMA: Camar, TILO: Tilomonte

In the *DAPC* analysis, three axes were retained and explained 94.2% of total variation (57.95%, 23.85%, and 12.40%, respectively; Figure 3). Considering the three scatterplots retrieved (Figure 3a–c), the populations are not very differentiated with the exception of ZAPI individuals that can be recognized as a separate group according to PC2. The PC1 versus PC2 scatterplot (Figure 3a) shows the ZAPI individuals separated from two groups partially differentiated integrated by the remaining populations. These overlapping groups can be partially associated with the sampling basin; one is integrated by CAMA, YAYE, TOCO, CHIU, and TILO, and a second group contains CANC, VVJO, TILI, QUIS, QUIN, QUIS, and TULO individuals. The TARA individuals are situated in an intermediate position according to PC1 and partially overlapped with both groups (Figure 3a,b). In PC1 versus PC3 scatterplot (Figure 3b), TULO and TARA individuals are relatively separated by PC3. The PC2 versus PC3 scatterplot (Figure 3c) reflects that the TULO individuals can also be considered partially differentiated.

### 3.3 Genetic structure among populations

**FIGURE 4:**
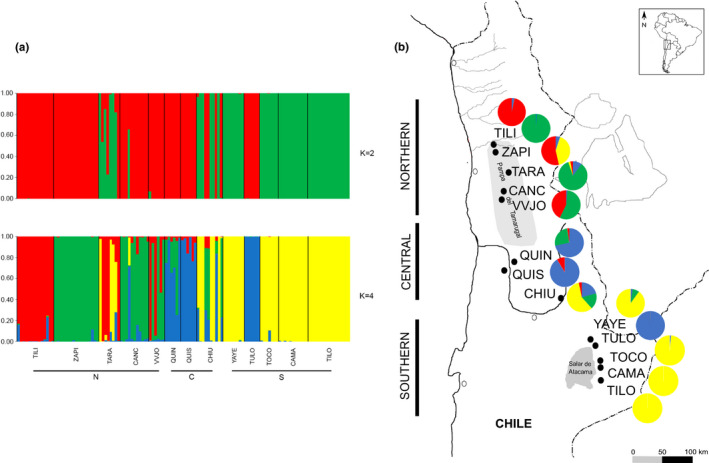
(a) Clustering of individuals for *K* = 2 and *K* = 4 based on STRUCTURE analysis considering *no‐admixture* model. Each individual is represented by a vertical bar that is partitioned into colored segments that represent the individual's estimated membership fractions. Same color in different individuals indicates that they are belonging to the same cluster. (b) Pie charts represent combined genetic ancestries of all individuals sampled in each population, as obtained from STRUCTURE for *K* = 4. The four different colors correspond to four different genetic clusters. TILI: Tiliviche, ZAPI: Zapiga, TARA: Tarapacá, CANC: Canchones, VVJO: Valle Viejo, QUIN: Quillagua Norte, QUIS: Quillagua Sur, CHIU: Chiu‐Chiu, YAYE: Yaye, TULO: Tulor, TOCO: Toconao, CAMA: Camar, TILO: Tilomonte. N: northern, C: central, S: southern

The STRUCTURE analysis revealed an optimal number of subpopulations at *K* = 2 by the Δ*K* criteria and *K* = 4 by the mean log‐likelihood of data for each value of *K* (Pritchard et al., 2000) in both no‐admixture and admixture models performed (Figure S1a,b, respectively). Consequently, we analyzed the structure distribution at *K* = 2 and 4 for both models (Figures 4 and S2, respectively).

According to Figure 4, with *K* = 2, the clusters retrieved are not related to geographic location. Individuals from TILI, ZAPI, CANC, VVJO, QUIN, QUIS, and TULO populations belong to the red cluster, and those from YAYE, TILO, CAMA, and TOCO to the green cluster, suggesting that these two population groups are genetically differentiated. TARA and CHIU populations would have individuals belonging to both genetic clusters.

For *K* = 4, individuals from TARA, CANC, VVJO, QUIN, QUIS, and CHIU exhibit admixture and are represented by clusters red, green, blue, and yellow. All the individuals from YAYE, TILO, CAMA, and TOCO are represented by the same genetic cluster (yellow). Individuals from TULO are represented by a genetic cluster (blue) that is absent in the southern basin and is represented only in the central population QUIN and QUIS. The individuals from TILI are represented by the red cluster and the individuals from ZAPI by the green one. These genetic groups are absent in the southern basin as they are only represented up to CHIU latitude.

### 3.4 Correlation between genetic and geographic distance

**FIGURE 5:**
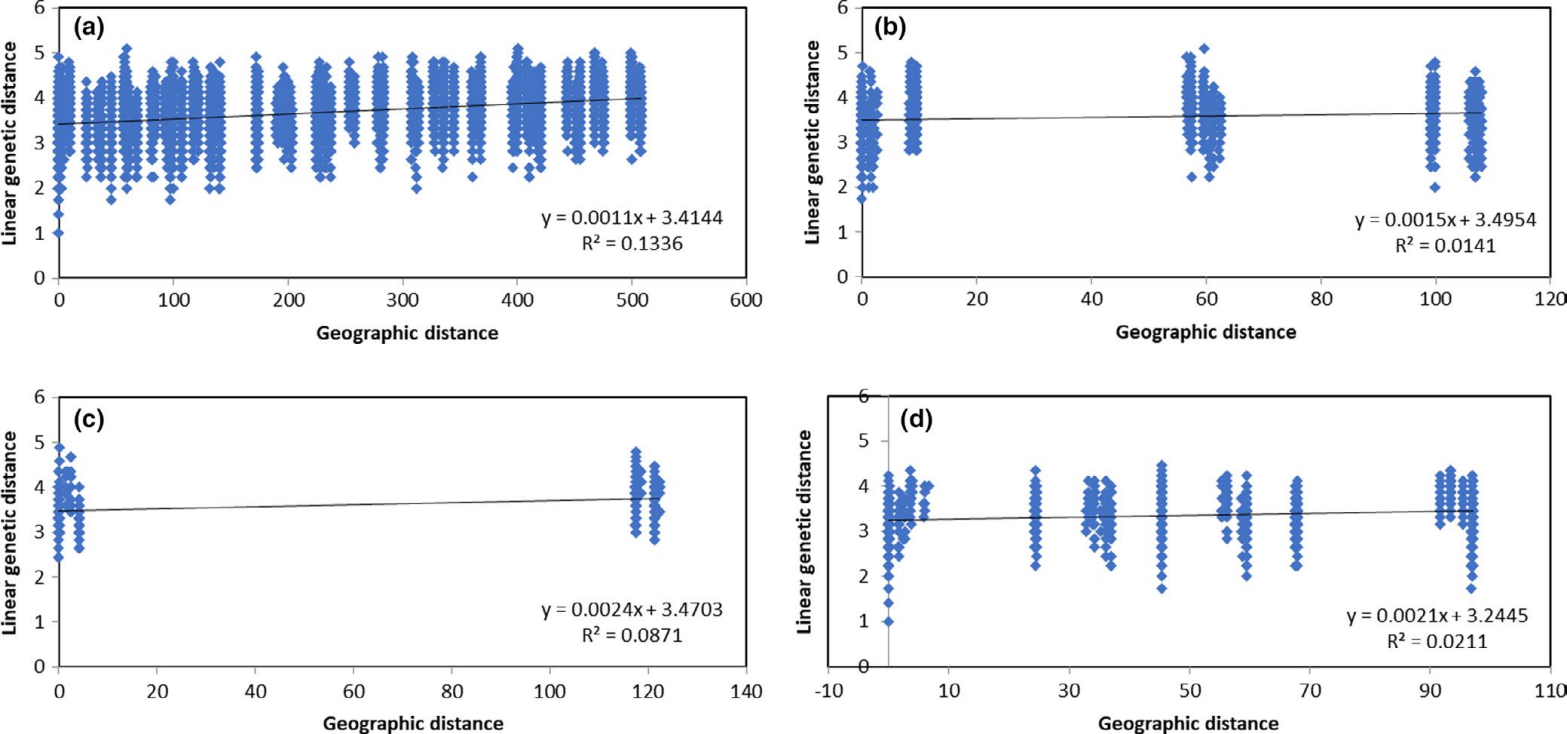
Pairwise genetic distances and geographical distances obtained from SSR considering all (a), the northern (b), the central (c), and the southern (d) populations studied in the Atacama Desert

We found significant correlation between genetic and geographical distances both when all sites were included (*r* = .37, *p* = .000) and when the analysis was performed considering the northern (*r* = .12, *p* = .020), central (*r* = .29, *p* = .001), and southern (*r* = .15, *p* = .000) basin populations separately (Figure 5). This result is consistent with the model of isolation by distance although the correlations obtained can be considered very low, especially within basins.

## 4 DISCUSSION

*Prosopis* populations in the Atacama Desert showed relatively high levels of genetic diversity (mean *H*_O_ = 0.61), comparable to the results from natural populations of *P. alba*, *P. flexuosa,* and *P. chilensis* (Section *Algarobia*) inside and outside Chile with different levels of isolation (Bessega et al., 2016, 2018; Moncada et al., 2019). These results are partially expected in the populations here studied, as individuals from these species are co‐occurring and the variability indices from these different species, based on SSR, are almost equal. Moreover, isozyme data yield similar genetic variability estimates and low genetic differentiation among species of Section *Algarobia* (Bessega et al., 2000; Saidman & Vilardi, 1987). As a consequence of geographic isolation, it is expected that genetic diversity within populations declines, associated with genetic drift or inbreeding (Bessega et al., 2018; Grivet et al., 2008). However, it may be that the geographic isolation consequences have not yet produced detectable effects on genetic diversity due to the long generation time of *Prosopis* species.

Genetic diversity can be spatially structured at different levels, such as landscape, population, or between nearby individuals, due to different ecological process of habitat characteristics such as population density and community structure operating in natural populations (Zeng et al., 2012). At a global level, *Prosopis* populations in the Chilean Desert show low but significant genetic differentiation (*F*_ST_ = 0.128, *F*_ST‐ENA_ = 0.129, and *D*_JOST_ = 0.238) and is consistent with the differentiation evaluated by the discriminant analysis of the principal components (*DAPC*), where differentiation is not observed at the uppermost hierarchical level as populations from northern, central, and southern basins were not grouped forming clusters. In agreement, low levels of variation were explained by AMOVA between basins (7.40%). Isolation between the studied basins can be attributed to ancient biogeographical processes linked to aridity fluctuations and geomorphological events that may have driven biological differentiation (Baranzelli et al., 2014; Ossa et al., 2013; Viruel et al., 2012). Significant differentiation patterns between the northern and southern groups of *P. chilensis* population in the Coquimbo Region (Chile) were attributed to differentiation dated more than 1 million years ago that has been blurred by more recent gene flow (Moncada et al., 2019). A regional examination of the Atacama Pacific Paleosurface (Evenstar et al., 2017) indicated that the basins here studied were established since ~8 to 3 Ma, and it is plausible to explain our results in a similar way, attributing that the ancient genetic differentiation between the northern, central, and southern basins has been blurred by more recent gene flow. Here, a relatively high number of migrants per generation were estimated at global level (*Nm* = 1.7); however, the paleo‐archeological evidences indicate that *Prosopis* species from Section *Algarobia* were not present in the Atacama Desert up to the Formative period (3,000–1,700 cal BP) ruling out so ancient possibility (McRostie et al., 2017; Ugalde et al., 2020).

When variation distribution is analyzed at lower hierarchical levels, most of the variation (79.3%) was found at the lowest level (individuals), which is expected in outcrossing species as *Prosopis* (Bessega et al., 2019; Chequer Charan et al., 2020; Roser et al., 2017). However, the AMOVA indicated that the genetic variation between populations within basins was low and significant (7.47%) and almost the same as the percentage obtained considering the between‐basin component (7.4%). But the migrants per generation estimations within basins were up to four times higher than the *Nm* estimate obtained for the total basins (*Nm* = 2.6, 6.9, and 2.5 within northern, central, and southern basins, respectively). Although Mantel's tests were also significant, indicating that nearby populations tend to be genetically more similar than expected by chance and suggesting gene flow restriction both at short and long distances, the low correlation estimates suggest a high degree of gene flow occurring within basins. According to these results, the geographic isolation that occurs between the populations within each of the basins seems to be able to be crossed by the natural dispersers of *Prosopis* easier than the large distances that separate the different basins considered here. These results are compatible with endozoic seed dispersal associated with short‐distance spread described for *Prosopis* (Burkart, 1976; Keys, 1993; Mares et al., 1977; Reynolds, 1954). Although native vectors for *Prosopis* have not been exhaustively studied in the area, these might be foxes, small rodents, and birds (Bessega et al., 2006; Burkart, 1976; Campos & Ojeda, 1997; Carevic et al., 2019; Maldonado et al., 2014). In arid zones of South America (southern Peru and northern Argentina), the diet of the fox (*Lycalopex culpaeus*) was reported to be comprised up to 69% of plants, and small quantity of *P. alba* seed plant remains in scats of *L. culpaeus* in the Atacama Desert (Carevic et al., 2019). Preliminary analyses being made on archaeological camelid coprolites (ca. 2,000 years BP) from several sites located in the Pampa del Tamarugal and Loa River basins do not show the preservation of entire seed of *algarrobo,* which, however, have been reported in archeological camelid dung in northern Peru (Shimada & Shimada, 1985).

The isolation by distance (IBD) detected both globally and within each basin can also be discussed in conjunction with the structure and pairwise differentiation results. Since many of the populations studied are constituted by more than one taxonomic species, it is possible to expect a high level of admixture. However, the level of admixture detected by the STRUCTURE analysis (both at *K* = 2 and *K* = 4) can be considered low. For *K* = 4, the populations with admixture signals were only six out of thirteen and are mainly located in the central basin (QUIN, QUIS, and CHIU) and in less proportion in the south of the northern basin (TARA, CANC, and VVJO). As predicted by the IBD model, neighbor populations are not expected to be more similar to each other than the distant ones. Here, we detected inconstancies in *F*_ST_, *F*_ST‐ENA_, and *D*_JOST_ differentiation indices among population based on a pairwise analysis considering the basins. Those inconsistencies were found in both the northern and southern basins. In the north, the ZAPI‐TILI and ZAPI‐TARA differentiation indices were comparatively higher than the ones detected within all the northern basin. Similarly, in the south, TULO individuals are much more differentiated from the remaining southern populations. Plausible causes that could explain the inconsistencies in pairwise *F*_ST_ estimations are genetic drift and non‐natural gene flow patterns. Small isolated populations are particularly susceptible to genetic drift and inbreeding, which are thought to reduce genetic diversity. The loss of genetic diversity in turn enhances inbreeding and finally results in inbreeding depression (Frankham et al., 2002). Here, no general trend to heterozygote excess or deficiency was detected and *F*_IS_ estimations fit the expectation for populations under the Hardy–Weinberg equilibrium. According to this, genetic drift does not appear to be the cause that explains the differentiation inconsistencies; however, it must be considered a possible source of error. The second plausible cause that could explain *F*_ST_, *F*_ST‐ENA_, and *D*_JSOT_ inconsistencies is the occurrence of non‐natural gene flow. Gene flow mediated by humans is a valid possibility and allows us to propose that in addition to natural dispersal, the transport of *algarrobo* pods/seeds may have been done by humans. Accelerator mass spectrometry dating of *algarrobo* endocarps throughout several archeological and paleoecological sites of the Chilean Atacama shows that trees from Section *Algarobia* appeared around 3,000 years before present as a consequence of human introduction together with other domesticated plants brought to the Atacama Desert (McRostie et al., 2017). The fact of having found various species belonging to the Section *Algarobia* in the Atacama oases and the SSR results, agree with the anthropic movement proposal, being possible that human movements explain the displacement of the fruits (Correa & García, 2014; Molina, 2017; Núñez, 1976; Núñez & Briones, 2017). Consequently, there are various species living together in the oases and an unnatural gene flow pattern is produced. The admixture pattern detected in the central basin is also compatible with its position within the Loa River course, which functioned as a sociocultural corridor throughout millennia. Quillagua oasis has been defined as a node of interregional interchange, whereas caravans and humans often passed in the transit between the coast and the highlands (Agüero et al., 2006; Briones, 2006; Berenguer & Pimentel, 2017; Cases & Montt, 2013; Gallardo, 2017; Martínez, 1998; Pimentel et al., 2017; Sanhueza, 1992). During historical times, the miner industry heavily disturbed the landscape especially within the northern basin. This industry reduced the use of *algarrobo* to coal and wood, but also to forage for European herbivores, especially mules which transit thoroughly from the coast to beyond the Andes (Carmona, 2018). These animals might have favored the encroachment of some species within oases (Brown & Archer, 1989); however, we cannot rule out that the patterns found were produced also in pre‐Columbian times, where a long history of interchange has been acknowledged from archaeological evidences (Uribe et al., 2020).

Historically, Atacama populations benefit from both *algarrobo* and *chañar (Geoffroea decorticans*) trees (Martínez, 1998). A recent study by Contreras et al. (2018) on the genetic structure of eight *Geoffroea decorticans* populations from the Atacama Desert has concluded that at least two different origins could explain the genomic differences in *chañar* populations in northern Chile. Similarly, if we consider the Δ*K* criteria, our structure results let us propose that two origins are also possible for *algarrobo* in Atacama (*K* = 2). A first approximation could be to link the two genetic groups and the altitude at which the different populations studied are found (Table 1). However, the simple observation of the altitudes and the genetic groups rules out this possibility. A plausible proposition to consider may be that northern basin may have received influence of populations from Bolivia and/or Peru, whereas southern and central samples could have been influenced by Argentinean populations despite the geographical Andes barrier. Although future studies may confirm this proposal, this interpretation is supported by archeological, ethnographical, and ethnohistorical data that show that in the Loa River and Salar de Atacama basin, people were interdigitated with communities from South Lipez (Bolivia Highlands), although communities from the *Salar the Atacama* basin were also connected and interdigitated with northwestern Argentina, especially from Toconao to the south (Hidalgo, 1995; Martínez, 1998).

In summary, we have assessed the genetic diversity of *Prosopis* populations (Section *Algarobia*) located in the oases of the Atacama Desert considering the genetic variability and its distribution, genetic differentiation, gene flow among populations, IBD patterns, and genetic structure. We have discussed the genetic patterns in reference to ecological, historical, and sociocultural characteristics. Based on the analyses of genetic parameters, we propose that the genetic pattern may be the consequence of natural gene flow together with anthropic transport of a wide variety of *algarrobo*.

## CONFLICT OF INTEREST

None.

## AUTHOR CONTRIBUTION

**Cecilia Bessega:** Conceptualization (equal); Formal analysis (equal); Funding acquisition (equal); Investigation (equal); Methodology (equal); Project administration (equal); Resources (equal); Software (equal); Supervision (equal); Validation (equal); Visualization (equal); Writing‐original draft (equal); Writing‐review & editing (equal). **Carolina Pometti:** Formal analysis (equal); Funding acquisition (equal); Methodology (equal); Resources (equal); Software (equal); Writing‐original draft (equal); Writing‐review & editing (equal). **Renee H Fortunato:** Data curation (equal); Resources (equal); Writing‐original draft (equal); Writing‐review & editing (equal). **Fransisca Greene :** Resources (equal); Writing‐original draft (equal); Writing‐review & editing (equal). **Calogero Santoro:** Funding acquisition (equal); Resources (equal); Supervision (equal); Writing‐original draft (equal); Writing‐review & editing (equal). **Virginia McRostie:** Conceptualization (equal); Funding acquisition (equal); Investigation (equal); Project administration (equal); Resources (equal); Supervision (equal); Writing‐original draft (equal); Writing‐review & editing (equal).

## Supporting information


**FIGURE S1**

**FIGURE S2**

**TABLES S1‐S3**

**Figure Legends**

## Data Availability

Sampling locations: Table 1. Microsatellite genotypic data will be submitted to Dryad and are available upon request (cecib@ege.fcen.uba.ar).
