# Supplementary figures and images for: Genetic studies of various Prosopis species (Leguminosae, Section Algarobia) co‐occurring in oases of the Atacama Desert (northern Chile)
Source: Ecol Evol. 2021 Feb 10;11(5):2375–90. doi: 10.1002/ece3.7212 (PMC7920779; doi:10.1002/ece3.7212)

A

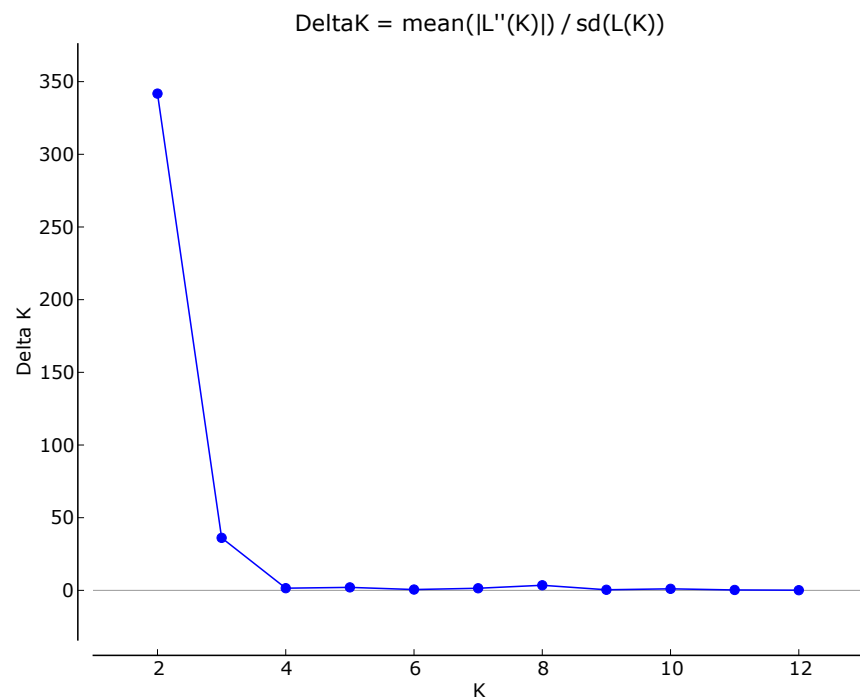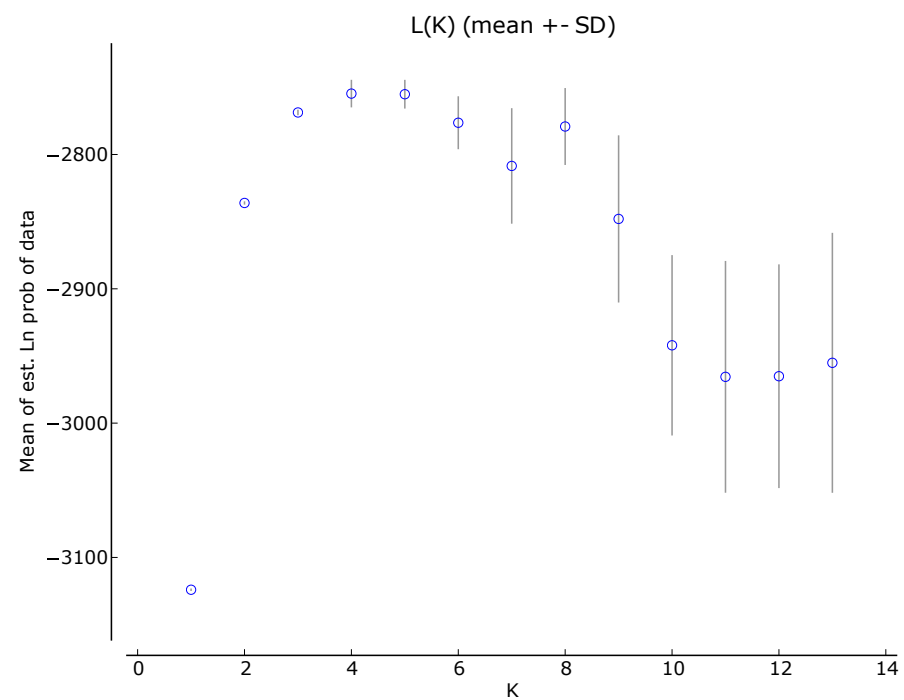

B

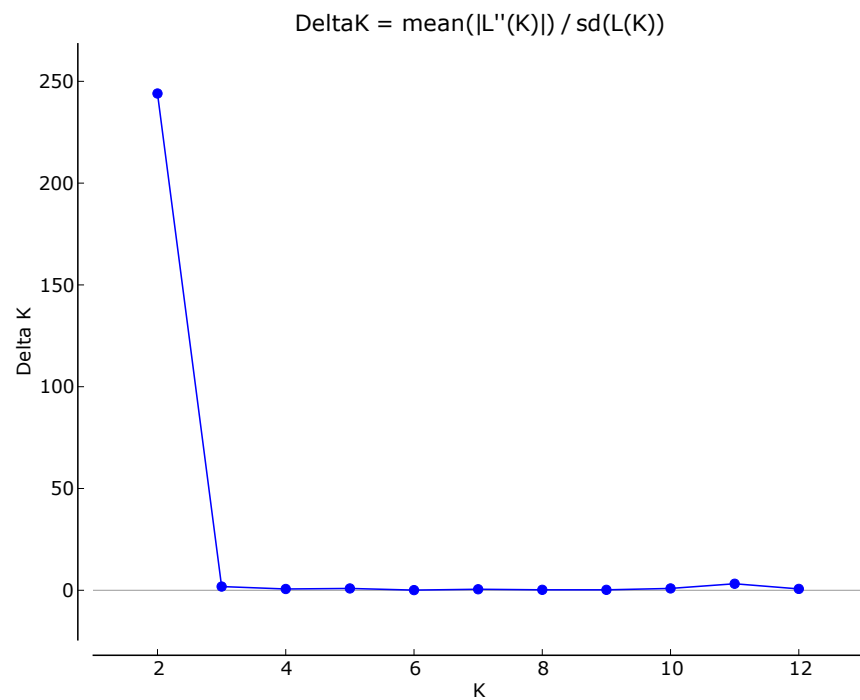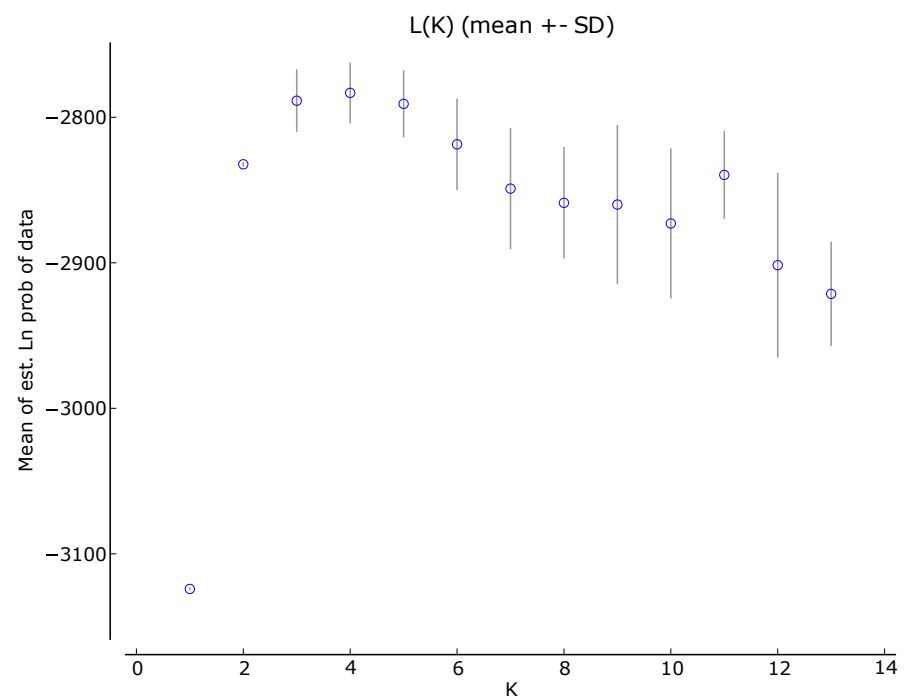

Supplement: Supplementary file 1 — FIGURE S1 [file ECE3-11-2375-s001.pdf]

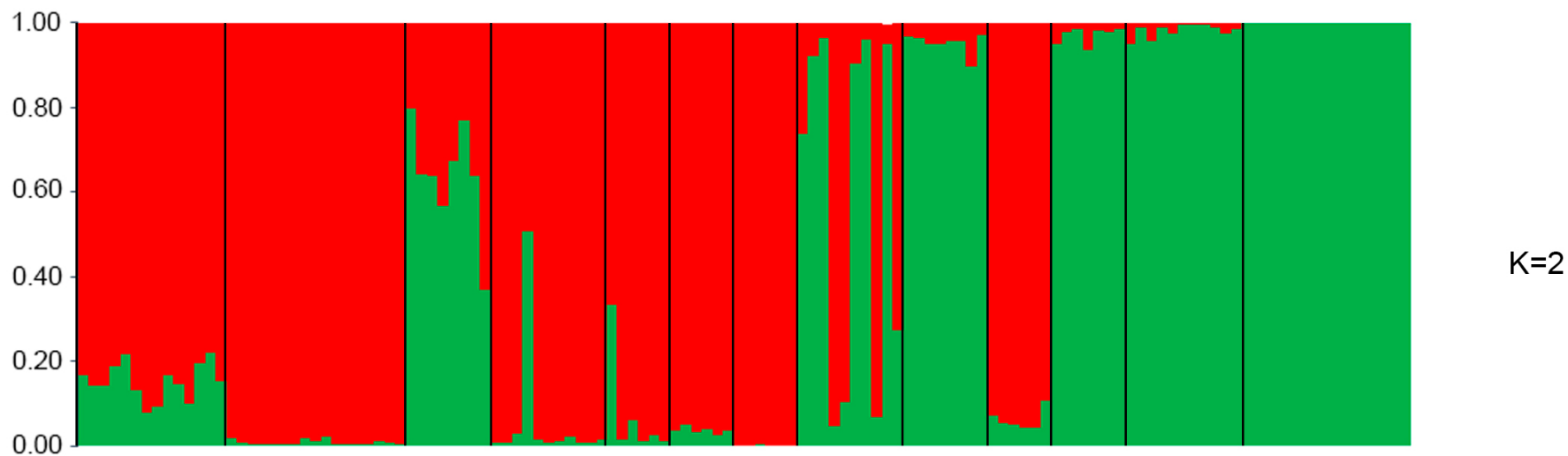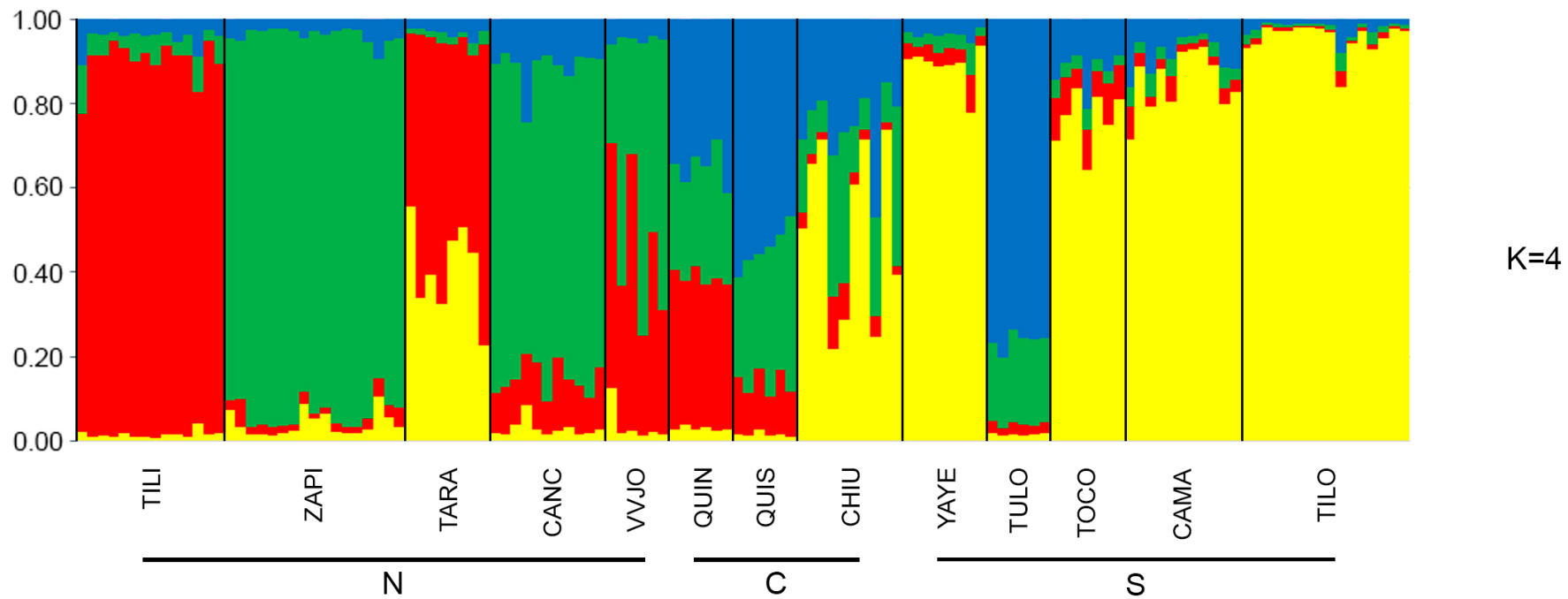

Supplement: Supplementary file 2 — FIGURE S2 [file ECE3-11-2375-s002.pdf]
