# Supplementary material for: Genetic studies of various Prosopis species (Leguminosae, Section Algarobia) co‐occurring in oases of the Atacama Desert (northern Chile)
Source: Ecol Evol. 2021 Feb 10;11(5):2375–90. doi: 10.1002/ece3.7212 (PMC7920779; doi:10.1002/ece3.7212)
Supplement: Supplementary file 3 — TABLES S1‐S3 [file ECE3-11-2375-s003.docx]

**Tables A1-A3.** Measures of genetic differentiation (*F_ST,_ F_ST-ENA_* and *D_JOST_*) in pairwise comparisons of the studied populations.

| **A1 -*F_ST_*** | | **N** | | | | | **C** | | | **S** | | | |
| --- | --- | --- | --- | --- | --- | --- | --- | --- | --- | --- | --- | --- | --- |
|  |  | **TILI** | **ZAPI** | **TARA** | **CANC** | **VVJO** | **QUIN** | **QUIS** | **CHIU** | **YAYE** | **TULO** | **TOCO** | **CAMA** |
| **N** | **ZAPI** | 0.096* |  |  |  |  |  |  |  |  |  |  |  |
|  | **TARA** | 0.047* | 0.108* |  |  |  |  |  |  |  |  |  |  |
|  | **CANC** | 0.055* | 0.060* | 0.075* |  |  |  |  |  |  |  |  |  |
|  | **VVJO** | 0.044 | 0.066* | 0.075* | 0.041 |  |  |  |  |  |  |  |  |
| **C** | **QUIN** | 0.047 | 0.068* | 0.064* | 0.048 | 0.046 |  |  |  |  |  |  |  |
|  | **QUIS** | 0.068* | 0.092* | 0.093* | 0.066* | 0.066 | 0.028 |  |  |  |  |  |  |
|  | **CHIU** | 0.073* | 0.089* | 0.055* | 0.056* | 0.073* | 0.057 | 0.078* |  |  |  |  |  |
| **S** | **YAYE** | 0.132* | 0.148* | 0.073* | 0.122* | 0.140* | 0.123* | 0.145* | 0.044 |  |  |  |  |
|  | **TULO** | 0.096* | 0.119* | 0.121* | 0.060* | 0.090* | 0.071* | 0.097* | 0.085* | 0.144* |  |  |  |
|  | **TOCO** | 0.109* | 0.138* | 0.079* | 0.119* | 0.129* | 0.103* | 0.130* | 0.052* | 0.046 | 0.120* |  |  |
|  | **CAMA** | 0.138* | 0.144* | 0.089* | 0.110* | 0.139* | 0.122* | 0.150* | 0.042 | 0.026 | 0.132* | 0.066* |  |
|  | **TILO** | 0.154* | 0.168* | 0.094* | 0.157* | 0.159* | 0.138* | 0.159* | 0.052* | 0.028 | 0.169* | 0.046* | 0.060* |

| **A2 - *F_ST-ENA_*** | | **N** | | | | | **C** | | | **S** | | | |
| --- | --- | --- | --- | --- | --- | --- | --- | --- | --- | --- | --- | --- | --- |
|  |  | **TILI** | **ZAPI** | **TARA** | **CANC** | **VVJO** | **QUIN** | **QUIS** | **CHIU** | **YAYE** | **TULO** | **TOCO** | **CAMA** |
| **N** | **ZAPI** | 0.148 |  |  |  |  |  |  |  |  |  |  |  |
|  | **TARA** | 0.037 | 0.150 |  |  |  |  |  |  |  |  |  |  |
|  | **CANC** | 0.066 | 0.080 | 0.076 |  |  |  |  |  |  |  |  |  |
|  | **VVJO** | 0.032 | 0.074 | 0.063 | 0.018 |  |  |  |  |  |  |  |  |
| **C** | **QUIN** | 0.040 | 0.078 | 0.055 | 0.031 | 0.015 |  |  |  |  |  |  |  |
|  | **QUIS** | 0.084 | 0.123 | 0.105 | 0.061 | 0.059 | 0.000 |  |  |  |  |  |  |
|  | **CHIU** | 0.092 | 0.121 | 0.033 | 0.055 | 0.070 | 0.045 | 0.072 |  |  |  |  |  |
| **S** | **YAYE** | 0.187 | 0.214 | 0.071 | 0.167 | 0.189 | 0.165 | 0.192 | 0.021 |  |  |  |  |
|  | **TULO** | 0.114 | 0.165 | 0.131 | 0.053 | 0.109 | 0.074 | 0.112 | 0.083 | 0.176 |  |  |  |
|  | **TOCO** | 0.157 | 0.205 | 0.084 | 0.169 | 0.175 | 0.137 | 0.175 | 0.037 | 0.022 | 0.145 |  |  |
|  | **CAMA** | 0.203 | 0.214 | 0.101 | 0.152 | 0.196 | 0.171 | 0.208 | 0.029 | 0.000 | 0.159 | 0.064 |  |
|  | **TILO** | 0.239 | 0.262 | 0.127 | 0.242 | 0.242 | 0.217 | 0.237 | 0.059 | 0.013 | 0.234 | 0.043 | 0.076 |

| **A3 -*D_JOST_*** | | **N** | | | | | **C** | | | **S** | | | |
| --- | --- | --- | --- | --- | --- | --- | --- | --- | --- | --- | --- | --- | --- |
|  |  | **TILI** | **ZAPI** | **TARA** | **CANC** | **VVJO** | **QUIN** | **QUIS** | **CHIU** | **YAYE** | **TULO** | **TOCO** | **CAMA** |
| **N** | **ZAPI** | 0.279* |  |  |  |  |  |  |  |  |  |  |  |
|  | **TARA** | 0.075* | 0.333* |  |  |  |  |  |  |  |  |  |  |
|  | **CANC** | 0.120* | 0.142* | 0.181* |  |  |  |  |  |  |  |  |  |
|  | **VVJO** | 0.054 | 0.136* | 0.155* | 0.02 |  |  |  |  |  |  |  |  |
| **C** | **QUIN** | 0.070* | 0.153* | 0.111* | 0.057 | 0.018 |  |  |  |  |  |  |  |
|  | **QUIS** | 0.132* | 0.222* | 0.215* | 0.109* | 0.085 | -0.066 |  |  |  |  |  |  |
|  | **CHIU** | 0.212* | 0.291* | 0.101* | 0.124* | 0.175* | 0.101 | 0.180* |  |  |  |  |  |
| **S** | **YAYE** | 0.392* | 0.466* | 0.144* | 0.361* | 0.429* | 0.365* | 0.414* | 0.044 |  |  |  |  |
|  | **TULO** | 0.223* | 0.307* | 0.317* | 0.089* | 0.183* | 0.121* | 0.185* | 0.200* | 0.373* |  |  |  |
|  | **TOCO** | 0.306* | 0.424* | 0.173* | 0.355* | 0.383* | 0.279* | 0.353* | 0.086* | 0.041 | 0.286* |  |  |
|  | **CAMA** | 0.437* | 0.467* | 0.233* | 0.328* | 0.445* | 0.382* | 0.456* | 0.054 | -0.013 | 0.343* | 0.128* |  |
|  | **TILO** | 0.460* | 0.522* | 0.239* | 0.496* | 0.490* | 0.418* | 0.451* | 0.105* | 0.007 | 0.445* | 0.065* | 0.118* |

N: northern, C: central, S: southern, TILI: Tiliviche, ZAPI: Zapiga, TARA: Tarapacá, CANC: Canchones, VVJO: Valle Viejo, QUIN: Quillagua Norte, QUIS: Quillagua Sur, CHIU: Chiu-Chiu, YAYE: Yaye, TULO: Tulor, TOCO: Toconao, CAMA: Camar, TILO: Tilomonte. For *F_ST_* and *D_JOST_* significant values are indicated with * based on 9999 permutations rand test
