# Supplementary material for: Genetic studies of various Prosopis species (Leguminosae, Section Algarobia) co‐occurring in oases of the Atacama Desert (northern Chile)
Source: Ecol Evol. 2021 Feb 10;11(5):2375–90. doi: 10.1002/ece3.7212 (PMC7920779; doi:10.1002/ece3.7212)
Supplement: Supplementary file 4 — Figure Legends [file ECE3-11-2375-s004.docx]

**Figure S1.** Plot of Δ(K) vs K and means probability of density values versus K *no-admixture* (A) and *admixture* (B) models.

**Notes:** Verticals bars indicate the confidence interval.

**Figure S2.** Clustering of individuals for K=2 and K=4 based on STRUCTURE analysis considering *admixture* model. Each individual is represented by a vertical bar that is partitioned into colored segments that represent the individual's estimated membership fractions. Same color in different individuals indicates that they are belonging to the same cluster.

**Notes:** TILI: Tiliviche, ZAPI: Zapiga, TARA: Tarapacá, CANC: Canchones, VVJO: Valle Viejo, QUIN: Quillagua Norte, QUIS: Quillagua Sur, CHIU: Chiu-Chiu, YAYE: Yaye, TULO: Tulor, TOCO: Toconao, CAMA: Camar, TILO: Tilomonte, N: northern, C: central, S: southern
